# The effect of the sex, age, and breed of farmed rabbits and the choice of management system on the extensity and intensity of *Eimeria* infection

**DOI:** 10.14202/vetworld.2020.1654-1660

**Published:** 2020-08-20

**Authors:** B. Pilarczyk, A. Tomza-Marciniak, R. Pilarczyk, E. Januś, P. Stanek, B. Seremak, P. Sablik

**Affiliations:** 1 Department of Animal Reproduction Biotechnology and Environmental Hygiene, Faculty of Biotechnology and Animal Husbandry, West Pomeranian University of Technology, Szczecin, Poland; 2 Department of Ruminant Science, Faculty of Biotechnology and Animal Husbandry, West Pomeranian University of Technology, Szczecin, Poland; 3 Laboratory for Organic Production of Food of Animal Origin, Institute of Animal Breeding and Biodiversity Conservation, University of Life Sciences in Lublin, Poland

**Keywords:** *Eimeria*, rabbits, small-scale rabbit farms

## Abstract

**Background and Aim:**

The most common causes of loss and diarrhea in rabbit farming are nutritional errors and coccidiosis. The infection can spread rapidly throughout a breeding area, reducing the rabbit population, and causing heavy losses. The aim of the study was to determine the influence of the system of animal management on the extensity and intensity of infection by Eimeria of farmed rabbits, together with the effect of the sex, age, and breed of the rabbits themselves.

**Materials and Methods:**

The study included 91 rabbits (Flemish Giant, New Zealand White, French Lope, Vienna Blue, California White, and mixed breed) from eight domestic (small-scale) farms from Poland. The prevalence and intensity of coccidial infection were determined by the Willis-Schlaf and McMaster coprological methods. The species were determined based on oocyst morphology: Their shape, color, form index, the presence or absence of micropyle and cap, and the presence or absence of residual, polar, and Stiedé bodies.

**Results:**

Seven species of Eimeria were isolated from the tested rabbits: Eimeria magna, Eimeria media, Eimeria perforans, Eimeria stiedae, Eimeria coecicola, Eimeria exigua, and Eimeria irresidua. Most infections were found to be of relatively low intensity. No significant differences in the extensity of Eimeria protozoan infection were observed with regard to sex. However, rabbit age had a significant influence on the extensity of infection by E. magna and of Eimerian protozoans combined. The greatest extensity was observed in rabbits aged below 6 months. For all species of Eimeria, greater extensity was observed among rabbits kept in groups than individually. The system of rabbit management also had a significant influence on the intensity of infection. Those kept in groups demonstrated a significantly higher mean intensity of infection of E. magna and all Eimeria species combined than those kept individually.

**Conclusion:**

Our findings indicate that Eimeria protozoa are a common occurrence on small-scale rabbit farms. As coccidiosis treatment does not always give good results, prevention is very important in the fight against this disease. It is necessary to develop a new preventive paradigm that pays special attention to the factors that promote the spread and development of infection in domestic (small-scale) farms from Poland. For example, it would be recommended to use large, dry, bright rooms with access to the sun, as these are conducive to preventing the occurrence of coccidia infections.

## Introduction

Rabbits are commonly farmed for their meat or hides; however, both juvenile and adult rabbits are subject to invasive diseases and parasite infections. Both can have varying influences on the health of the rabbits and the cost-effectiveness of their farming, and are responsible for the greatest losses. The scale of the problem depends to a certain degree on the intensity of farming, with rabbits kept at high densities being more seriously affected than those kept individually [1,2].

The most common causes of loss and diarrhea in rabbit farming are nutritional errors and coccidiosis; the latter of which is caused by protozoa of the genus *Eimeria*. Coccidiosis can occur in intestinal or hepatic forms, and can quickly spread, thanks to the direct and short developmental cycle of coccidians [3]. Young rabbits are the most susceptible to infection, particularly those immediately after weaning, while adult rabbits are often asymptomatic carriers of *Eimeria*. Clinical coccidiosis is characterized by apathy, chronic, or acute diarrhea, dehydration and reduced weight gain due to poor food use and decreased appetite, often resulting in death [4]. The infection can spread rapidly throughout a breeding area, reducing the rabbit population, and causing heavy losses.

The majority of rabbits kept commercially in Poland are reared on domestic (small-scale) farms. However, as such fragmentation is typically more prone to biosecurity errors, such systems are at higher risk of the parasite infection. Hence, to minimize the threat of such infection, it is important to use correct biosecurity procedures and to design and implement appropriate prophylactic programs.

The aim of the study was to determine the influence of the system of animal management on the extensity and intensity of infection by *Eimeria* of farmed rabbits, together with the effect of the sex, age, and breed of the rabbits themselves.

## Materials and Methods

### Ethical approval

The tests were performed on feces samples taken from farmed rabbits. Under Polish law, this study was exempt from the need to obtain Local Ethics Committee approval.

### Animals

The study was conducted from September to November 2019 and included 91 rabbits (Flemish Giant, New Zealand White, French Lope, Vienna Blue, California White, and mixed breed) from eight domestic (small-scale) farms from Poland. The rabbits were kept in cages stored in the open air, under a roof or indoors. The floors of the cages consisted of wooden grates or welded mesh. The rabbits were fed farm fodder consisting of cereals (barley, wheat, and oats), bran, dry roughage (hay), and root crops (carrots, beetroots, and steamed potatoes) as well as green fodder.

### Fecal samples

Feces samples were collected from each animal. For this purpose, plastic sheets were placed under the floor of the cages. Individual fecal samples were collected in plastic bags, transported to the laboratory, and stored at 4°C until analysis.

### Parasitological analysis

The prevalence and intensity of coccidial infection were determined by the Willis-Schlaf and McMaster coprological methods [5].

The species were determined based on oocyst morphology: Their shape, color, form index, the presence or absence of micropyle and cap, and the presence or absence of residual, and polar and Stiedé bodies. Identification was also performed based on their time of sporulation, facilitated in a wet chamber at 24-26°C in a 2.5% aqueous solution of potassium dichromate (K_2_Cr_2_O_7_) [6].

### Statistical analysis

Statistical analysis was performed using Statistica 13.3 software (TIBCO Software Inc., Palo Alto, CA, USA). The influence of selected factors (gender, age, race, housing system, and farm) on the extensity of *Eimeria* infection was determined with the χ^2^ test. In addition, the significance of the intensity of infection was evaluated with the Mann–Whitney or Kruskal–Wallis test.

## Results

Seven species of *Eimeria* were isolated from the tested rabbits: *Eimeria magna, Eimeria media, Eimeria perforans, Eimeria stiedae*, *Eimeria coecicola*, *Eimeria exigua*, and *Eimeria irresidua*. Detailed data on the extensity of the infection with regard to individual species of *Eimeria* are presented in Table-1. Most infections were found to be of relatively low intensity (Table-2).

**Table-1:** Extensity of infection (%) of rabbits with *Eimeria* spp. with regard to sex, age, breed, and system of management.

Factors	*Eimeria magna*		*Eimeria media*		*Eimeria perforans*		*Eimeria stiedae*		*Eimeria coecicola*		*Eimeria exigua*		*Eimeria irresidua*		Combined	
							
	A/B	E.I.	A/B	E.I.	A/B	E.I.	A/B	E.I.	A/B	E.I.	A/B	E.I.	A/B	E.I.	A/B	E.I.
Sex																
♂	13/20	65.0	14/20	70.0	7/20	35.0	3/20	15.0	4/20	20.0	3/20	15.0	5/20	25.0	14/20	70.0
♀	52/71	73.2	43/71	60.6	20/71	28.2	12/71	16.9	11/71	15.5	9/71	12.7	12/71	16.9	54/71	76.1
	χ^2^=0.5; p=0.47		χ^2^=0.6; p=0.44		χ^2^=0.4; p=0.55		χ^2^=0.1; p=0.84		χ^2^=0.2; p=0.63		χ^2^=0.1; p=0.79		χ^2^=0.7; p=0.41		χ^2^=0.3; p=0.58	
Age (months)																
<6	28/33	84.9	23/33	69.7	10/33	30.3	8/33	24.2	6/33	18.2	2/33	6.1	7/33	21.2	28/33	84.9
6-12	12/22	54.6	10/22	45.5	6/22	27.3	2/22	9.1	2/22	9.1	2/22	9.1	3/22	13.6	12/22	54.6
>12	25/36	69.4	24/36	66.7	11/36	30.6	5/36	13.9	7/36	19.4	8./36	22.2	7/36	19.4	28/36	77.8
	χ^2^=6.0; p=0.04		χ^2^=3.7; p=0.15		χ^2^=0.1; p=0.96		χ^2^=2.5; p=0.29		χ^2^=1.2; p=0.55		χ^2^=4.4; p=0.11		χ^2^=0.5; p=0.77		χ^2^=6.7; p=0.03	
Breed																
Flemish Giant	17/22	77.3	14/22	63.6	3/22	13.6	3/22	13.6	2/22	9.0	0/22	0	3/22	13.6	17/22	77.3
New Zealand White	18/18	100	15/18	83.3	6/18	33.3	4/18	22.2	4/18	22.2	5/18	27.8	6/18	33.3	18/18	100
French Lope	8/8	100	8/8	100	3/8	37.5	1/8	12.5	2/8	25.0	2/8	25.0	1/8	12.5	8/8	100
Vienna Blue	1/4	25.0	1/4	25.0	1/4	25.0	1/4	25	0/4	0	0/4	0	1/4	25.0	1/4	25
California White	1/3	33.3	0/3	0	0/3	0	0/3	0	0/3	0	0/3	0	0/3	0	1/3	33.3
Mixed breed	20/36	55.6	19/36	52.8	14/36	38.9	6/36	16.7	7/36	19.4	5/36	13.9	6/36	16.7	23/36	63.9
	χ^2^=21.6; p<0.001		χ^2^=17.0; p<0.001		χ^2^=5.8; p=0.22		χ^2^=1.4; p=0.86		χ^2^=3.3; p=0.68		χ^2^=8.7; p=0.04		χ^2^=4.0; p=0.51		χ^2^=19.7; p<0.001	
System of management																
Individual	26/52	50.0	22/52	42.3	14/52	26.9	4/52	7.7	7/52	13.5	4/52	7.7	6/52	11.5	29/52	55.8
Group	39/39	100	35/39	89.7	13/39	33.3	11/39	28.2	8/39	20.5	8/39	20.5	11/39	28.2	39/39	100
	χ^2^=27.3; <0.001		χ^2^=21.4; <0.001		χ^2^=0.4; p=0.51		χ^2^=6.8; p<0.01		χ^2^=0.8; p=0.37		χ^2^=3.2; p=0.07		χ^2^=4.1; p=0.04		χ^2^=31.5; <0.001	

Legend: E.I.=Extensity of infection, A/B=Infected/examined

**Table-2:** Infection intensity (OPG) of rabbits with *Eimeria* spp. depending on gender.

Species	♂				♀				Mann–Whitney test
	
	n	GM	Me	Min.-Max.	n	GM	Me	Min.-Max.	
Protozoa of the genus *Eimeria*									
*Eimeria magna*	13	4348	5000	500-15,500	52	5221	6000	500-26,000	Z=0.77; p=0.44
*Eimeria media*	14	2215	4000	200-13500	43	2708	3500	100-15,000	Z=0.28; p=0.78
*Eimeria perforans*	7	610	500	500-1000	20	1333	2000	500-5000	Z=2.17; p=0.03
*Eimeria stiedae*	3	630	500	500-1000	12	652	500	500-3000	U=17.0; p=0.94
*Eimeria coecicola*	4	1800	1850	1000-3500	11	730	500	500-2000	U=4.0; p=0.02
*Eimeria exigua*	3	1339	1000	800-3000	9	726	500	100-3000	U=8.0; p=0.36
*Eimeria irresidua*	5	1084	500	500-6000	12	802	500	500-2000	U=26.5; p=0.75
Total *Eimeria* spp.	14	8982	9250	1000-35,500	54	9076	10,000	500-35,500	Z=0.21; p=0.83

OPG=Number of oocysts per gram of feces, n=Number of infected animals, GM=Geometric mean, Me=Median

No significant differences in the extensity of *Eimeria* protozoan infection were observed with regard to sex. However, rabbit age had a significant influence on the extensity of infection by *E. magna* (χ^2^=6.0; p=0.04) and of *Eimerian* protozoans combined (χ^2^=6.7; p=0.03). The greatest extensity was observed in rabbits aged below 6 months (Table-1).

The breed of the farmed rabbits significantly influenced the spread of *E. magna* (χ^2^=21.6; p<0.001), *E. media* (χ^2^=17.0, p<0.001), *E. exigua* (χ^2^=8.7; p=0.04), and of all the protozoan combined (χ^2^=19.7; p<0.001). The highest extensity was observed in New Zealand White and French Lope rabbits, and the lowest in Vienna Blue and Californian White; however, this may be due to the low number of individuals of the latter two. The system of animal management had a significant influence on the extensity of infection by *E. magna* (χ^2^=27.3; p<0.001), *E. media* (χ^2^=21.4; p<0.001), *E. stiedae* (χ^2^=6.8; p<0.01), *E. irresidua* (χ^2^=4.1; p=0.04), and all protozoa combined (χ^2^=31.5; p<0.001). For all species of *Eimeria*, greater extensity was observed among rabbits kept in groups than individually (Table-1).

Significant differences in the mean intensity of *E. perforans* and *E. coecicola* infection were observed between males and females. A significantly higher intensity of *E. perforans* infection (Z=2.17; p=0.03) was found among the females, and *E. coecicola* infection among the males (U=4.0; p=0.02) (Table-2).

Significant differences in the intensity of infection were observed between the tested age groups for *E. magna* and combined *Eimeria* infection. Both demonstrated significantly higher intensity (p≤0.05) among rabbits aged <6 months compared to those aged 6-12 months (Table-3).

**Table-3:** Infection intensity (OPG) of rabbits with *Eimeria* spp. with regard to age.

Species	<6 months				6-12 months				>12				Kruskal–Wallis test
		
	n	GM	Me	Min.-Max.	n	GM	Me	Min.-Max.	n	GM	Me	Min.-Max.	
*Eimeria* spp. protozoa													
*Eimeria magna*	28	7104^A^	9000	500-26,000	31	3913^A^	5000	500-20,000	6	3703	4250	500-12,500	H=9.4; p=0.01
*Eimeria media*	23	2347	3500	100-8500	28	2853	5000	200-15000	6	2299	3000	500-13,500	H=0.58; p=0.75
*Eimeria perforans*	10	1320	2000	500-4000	12	824	500	500-2500	5	1443	2000	500-5000	H=2.09; p=0.35
E. stiedae*	8	744	500	500-3000	6	500	500	500-500	1	1000	1000	1000-1000	
E. coecicola*	6	794	1000	500-1000	8	923	750	500-3500	1	2500	2500	2500-2500	
E. exigua*	2	707	750	500-1000	8	740	650	100-3000	2	1732	2000	1000-3000	
*Eimeria irresidua*	7	869	500	500-2000	7	713	500	500-2000	3	1442	1000	500-6000	H=1.17; p=0.55
Combined *Eimeria* spp.	28	11541^a^	14750	1000-29,000	33	7406^a^	8500	500-35,500	7	8863	7500	2500-35,500	H=5.83; p=0.05

* Due to the very small number of individuals infected with *E. stiedae*, *E. coecicola* and *E. exigua*, they were excluded from the statistical tests.

A =The same upper case letters denote statistically significant differences at p<0.01,

a =The same lower case letters denote statistically significant differences at p<0.05, OPG=Number of oocysts per gram of feces. n=Number of infected animals, GM=Geometric mean, Me=Median,* E. stiedae=Eimeria stiedae, E. coecicola=Eimeria coecicola, E. exigua=Eimeria exigua*

Similarly, the intensity of infection by *E. media* and total *Eimeria* was significantly influenced by the breeds of rabbit. The Flemish Giant and New Zealand White rabbits demonstrated significantly higher mean intensity of *E. media* infection than the French Lope and mixed breeds (p≤0.05), while the New Zealand Whites displayed significantly higher intensity of infection for all *Eimeria* combined than the mixed breeds (p≤0.05). The Vienna Blue and California White specimens infected with *E. stiedae*, *E. coecicola*, or *E. exigua* were not included in the statistical analysis due to the very small number of specimens (Table-4).

**Table-4:** The intensity of *Eimeria* infection (OPG) of the tested rabbits according to breed.

Species	Flemish giant				New Zealand white				French lope				Mixed breed				Kruskal-Wallis test
			
	n	GM	Me	Min.-Max.	n	GM	Me	Min.-Max.	n	GM	Me	Min.-Max.	n	GM	Me	Min.-Max.	
Protozoa of the genus *Eimeria*																	
*Eimeria magna*	17	7036	8000	2500-15,500	18	6339	5500	2500-20,000	8	4648	6000	500-15,500	20	3630	3750	500-26,000	H=3.53; p=0.32
*Eimeria media*	14	4557^ad^	4250	1000-8500	15	4382^bc^	5000	500-15,000	8	1249^cd^	750	500-13,500	19	1500^ab^	2000	100-9000	H=16.0; p=0.001
*Eimeria perforans*	3	1587	2000	500-4000	6	1710	2000	500-2500	3	630	500	500-1000	14	983	750	500-5000	H=4.38; p=0.22
Eimeria stiedae*	3	909	500	500-3000	4	595	500	500-1000	1	1000	1000	1000-1000	6	561	500	500-1000	
E. coecicola*	2	707	750	500-1000	4	1368	1500	500-3500	2	1581	1750	1000-2500	7	691	500	500-1200	
E. exigua*	0				5	1292	1000	500-3000	2	1732	2000	1000-3000	5	416	500	100-1000	
*Eimeria irresidua*	3	1145	1500	500-2000	6	757	500	500-2000	1	6000	6000	6000-6000	6	707	500	500-2000	H=4.20; p=0.24
Combined *Eimeria* spp.	17	11972	12500	4000-20,500	18	13102^a^	12400	7000-35,500	8	7644	8500	1000-35,500	23	6676^a^	6500	500-29,000	H=9.52; p=0.02

* Due to the very small number of individuals infected with *E. stiedae*, *E. coecicola,* and *E. exigua*, they were excluded from the statistical tests. ^a, b, c, d^=The same lower case letters denote statistically significant differences at p<0.05, OPG=Number of oocysts per gram of feces. n=Number of infected animals, GM=Geometric mean, Me=Median,

E. stiedae=Eimeria stiedae, E. coecicola=Eimeria coecicola, E. exigua=Eimeria exigua

The system of rabbit management also had a significant influence on the intensity of infection. Those kept in groups demonstrated a significantly higher mean intensity of infection of *E. magna* and of all *Eimeria* combined than those kept individually (p<0.001) (Table-5).

**Table-5:** Infection intensity (OPG) of rabbits with *Eimeria* spp. according to the system of animal management.

Species	Individual				Group				Mann–Whitney test
	
	n	GM	Me	Min.-Max.	n	GM	Me	Min.-Max.	
*Eimeria magna*	26	3013	3250	500-21,000	39	7087	8000	500-26,000	Z=4.30; p<0.001
*Eimeria media*	22	1915	3000	100-6000	35	3107	3500	500-15,000	Z=1.59; p=0.11
*Eimeria perforans*	14	1068	1000	500-4000	13	1111	1000	500-5000	Z=0.13; p=0.90
*Eimeria stiedae*	4	595	500	500-1000	11	667	500	500-3000	U=21.0; p=0.95
*Eimeria coecicola*	7	1148	1000	500-3500	8	771	750	500-2000	U=18.0; p=0.27
*Eimeria exigua*	4	707	750	500-1000	8	926	900	100-3000	U=13; p=0.67
*Eimeria irresidua*	6	561	500	500-1000	11	1117	1500	500-6000	U=17.5; p=0.13
All *Eimeria* spp.	29	5816	7000	500-24,600	39	12588	16000	1000-35,500	Z=4.50; p<0.001

OPG=Number of oocysts per gram of feces, n=Number of infected animals, GM=Geometric mean, Me=Median

## Discussion

The conditions of animal management have a significant influence on the state of infection of farmed rabbits and the course of parasite infestation [7-9]. Parasites have been found to be present in rabbits, even under laboratory conditions, where biosecurity measures are applied strictly [10].

A diagnosis of coccidiosis is confirmed by the presence of a very large number of *Eimeria* oocysts following stool examination. However, it is not unusual to identify a small number of oocysts in rabbit feces during the microscopic examination, and their presence does not confirm the presence of disease. In addition, number of oocysts excreted by the host is not always correlated with the presence of disease. This is due to the different pathogenicity of individual *Eimeria* species. The pace of coccidiosis depends primarily on the age of the animal and the virulence of the coccidia species. This is confirmed by our present observations, which revealed no clinical symptoms of disease in rabbits with high-intensity infections.

*E. stiedae* was found in 16.48% of the tested rabbits and *E. perforans* in 29.67%. These species are particularly dangerous for rabbits. *E. perforans* is known to be responsible for intestinal coccidiosis, characterized by seizures, paralysis, and the subsequent death of the animal, while *E. stiedae* has been linked to hepatic coccidiosis which, in severe cases, damages the liver, and bile ducts, resulting in death [8,9,11,12].

The extensity of infection for all *Eimeria* combined was found to be 74.72%; however, some variation has been recorded in previous studies. Ilić *et al*. [13] reported an *Eimeria* extensity of 50.65%, while Elshahawy and Elgoniemy [14] reported half our observed extensity among rabbits in Egypt (33.9%). These differences were most probably related to differences in environmental conditions, such as temperature and humidity. Even so, Szkucik *et al*. [15] reported similar findings to ours among rabbits in Poland (78.83%), but with intestinal coccidiosis dominating (56.5%) and significantly greater extensity of *E. stiedae* infection (3.34%). Similarly, Abdel-Baki and Al-Quraishy [16] reported 75.0% extensity of *Eimeria* in a Saudi Arabian study, and Nosal *et al*. [17] noted values ranging from 72.0 to 89.6%; however, the latter also indicated lower mean oocyst intensity than in the present study (487 to 2402 OPG).

A significant difference was found in the infection intensity of *E. perforans* and *E. coecicola* between male and female rabbits. These findings confirm those of Pakandl *et al*. [18] and Papeschi *et al*. [19], who also reported that the number of oocysts present in feces is correlated with the age, sex, and state of health of the host rabbit.

The age of the rabbit also appears to have a significant influence on the extensity of infection in this study. The greatest extensity was found in rabbits under 6 months of age, and only occasional infection was observed among those aged <3 weeks. Pakandl and Hlásková [20] reported no infection with *Eimeria* among sucking rabbits younger than 19 days. Coccidiosis typically affects younger rabbits aged 5-6 weeks, immediately after weaning, when the young rabbits possess low resistance to infection. The weaning phase is hence a critical period and may lead to significant losses. In addition, the strength of infection intensifies mainly during the perinatal period and before weaning, which is associated with a considerable decrease in immunity. Older rabbits are typically carriers of coccidians [17,21-25]. Similarly, Ilić *et al*. [13] reported a greater extensity of coccidial infection in young rabbits (50.6%) than older ones (37.6%).

The system of management also has a significant influence on the extensity of *Eimeria* infection, particularly *E. magna*, *E. media*, *E. stiedae*, and *E. irresidua*. Significantly greater extensity of infection was observed among rabbits kept in groups than those kept individually. In addition, the rabbits kept in group’s also demonstrated greater mean intensity of infection by *E. magna*, as well as by *Eimeria* in general. Similarly, Okumu *et al*. [26] also reported higher infection intensity in rabbits kept in groups and in multi-level cages. Kornaś *et al*. [27] noted that it is possible to reduce the occurrence of parasites in rabbits kept in cage system; however, this is not the case for *Eimeria* protozoans due to their short developmental cycle. Sadzikowski *et al*. [28] reported a significantly higher number of oocysts per gram of feces (OPG) in rabbits kept on individual properties than those kept on breeding farms.

Rabbits with age below 6 months demonstrated significantly higher mean intensity of infection by *E. magna* and all *Eimeria* combined than those aged 6-12 months. This is probably due to the fact that different species of rabbit coccidia parasitize different sections of the intestine, and hence the immune response to infection may differ between them [17]. *E. perforans, E. media*, and *E. irresidua* induce changes in the duodenum and jejunum, *E. magna* and *E. intestinalis* in the iliac gut, and *E. flavescens* in the cecum and colon. In rabbits, OPG values below 2000 oocysts per gram of feces are considered safe [29]. Varga [30] listed *E. intestinalis, E. flavescens*, and *E. stiedae* as the most pathogenic species, *E. magna, E. irresidua*, and *E. piriformis* as pathogenic and *E. perforans, E. coecicola*, and *E. media* as the least pathogenic.

On small-scale farms, coccidiosis can be controlled by the systematic replacement of litter, keeping cages dry, avoiding excessive density of rabbits, using age-rearing systems, as well as by maintaining appropriate temperature and humidity in the cages, and isolating sick individuals. However, *Eimeria* is resistant to changes in environmental conditions and disinfectants, which almost makes it impossible to completely eliminate them; therefore, cages and water and feed containers should also be regularly cleaned and disinfected, and the feces of rabbits should be regularly checked for the presence of parasites.

## Conclusion

Our findings indicate that *Eimeria* protozoa are a common occurrence on small-scale rabbit farms. As the coccidiosis treatment does not always give good results, prevention is very important in the fight against this disease. It is necessary to develop a new preventive paradigm that pays special attention to the factors that promote the spread and development of infection in this type of farm. For example, it would be recommended to use large, dry, and bright rooms with access to the sun, as these are conducive to preventing the occurrence of coccidia.

## Authors’ Contributions

BP designed the concept of research, performed the parasitological analysis, drafted the manuscript, analyzed, and interpreted data. AT designed the concept of research, drafted the manuscript, and revised the manuscript. RP designed the concept of research, performed the statistical analysis of all data. EJ designed the concept of research and coordinated the collection of the samples. PS, BS, and PSa collected the samples and data. All authors read and approved the final manuscript.

## Competing Interests

The authors declare that they have no competing interests.

## Publisher’s Note

Veterinary World remains neutral with regard to jurisdictional claims in published institutional affiliation.
